# Synergistic antitumor effects of circularly permuted TRAIL with doxorubicin in triple-negative breast cancer: Circularly permuted TRAIL with Dox in TNBC

**DOI:** 10.3724/abbs.2023160

**Published:** 2023-08-10

**Authors:** Jia Liu, Tienian Zhu, Jiankun Liu, Yujie Cui, Shifang Yang, Ruijing Zhao

**Affiliations:** 1 Department of Oncology Hebei Medical University Shijiazhuang 050017 China; 2 Department of Hematology Affiliated Hospital of Hebei University Baoding 071000 China; 3 Department of Medical Oncology Bethune International Peace Hospital Shijiazhuang 050082 China; 4Department of Oncology Hebei General Hospital Shijiazhuang 050057 China; 5 Beijing Sunbio Biotech Co. Ltd. Beijing 100000 China; 6Department of Immunology Hebei Medical University Key Laboratory of Immune Mechanism and Intervention on Serious Disease in Hebei Province Shijiazhuang 050017 China

**Keywords:** apoptosis, TRAIL, doxorubicin, death receptor, triple-negative breast cancer

## Abstract

Circularly permuted TRAIL (CPT), a novel recombinant TRAIL mutant, is a potent antitumor agent. However, its efficacy in triple-negative breast cancer (TNBC) remains unclear. Treatment with CPT alone and in combination with doxorubicin (Dox) is explored for its effects on the proliferation and apoptosis of MDA-MB-231 (MB231) and MDA-MB-436 (MB436) breast cancer cells *in vitro* and *in vivo*. Here, we show that CPT combined with Dox exhibits time- and dose-dependent synergy to inhibit cell viability and enhance apoptosis of MB231 and MB436 cells. Combined treatment substantially increases caspase-8, caspase-3, and PARP cleavage in both cell lines and significantly suppresses tumor growth in nude mice bearing MB231 xenografts. Collectively, our findings demonstrate that treatment with CPT in combination with Dox exerts synergistic antitumor effects through activation of the caspase cascade pathway, a mechanism that is partly dependent on the Dox-induced upregulation of death receptor 4 and death receptor 5. Therefore, CPT combined with Dox may be a feasible therapeutic strategy for the management of TNBC.

## Introduction

Breast cancer is the most prevalent type of cancer and shows the highest morbidity among women, 15% of which is triple-negative breast cancer (TNBC). TNBC has no expression of estrogen/progesterone receptor or human epidermal growth factor receptor-2 (HER-2) [ 1, 2] . Due to the lack of these targetable receptors, patients with TNBC have difficulty in benefiting from targeted therapies [2]. Currently, doxorubicin (Dox) remains an important drug for breast cancer treatment. The main adverse reaction to Dox is cardiotoxicity, and the dose is proportional to the incidence of heart failure, which limits its cumulative dose [3]. Dox is often used together with cyclophosphamide and paclitaxel to treat breast cancer [2]. Although the efficacy is enhanced when using chemotherapy drugs in combination, this also increases toxicity and side effects. Hence, there is a pressing need to develop a combination therapy strategy with high efficiency and low toxicity for breast cancer, especially TNBC.

Tumor necrosis factor-related apoptosis-inducing ligand (TRAIL) is selectively toxic to various cancer cells, including TNBC cells, by engaging death receptor 4 (DR4) and DR5 while sparing normal cells [4]. However, TRAIL has struggled clinically, likely due to inadequate target engagement [5]. Circularly permuted TRAIL (CPT) is a novel agent candidate in antineoplastic therapy and is an allosteric form of wild-type TRAIL. It displays excellent stability and biological activity and a better antitumor effect than wild-type TRAIL in lung, colorectal, and breast cancer cells [6]. In multiple myeloma preclinical studies and phase II clinical trials, CPT has shown good safety, tolerability and efficacy as a single, combined targeted and chemotherapy agent [ 7, 8] . However, the efficacy of circularly permuted TRAIL (CPT) in combination with Dox for TNBC is unknown to date.

In this study, we probed the synergistic antineoplastic activity of CPT in combination with Dox and the potential mechanisms using two TNBC cell lines, MDA-MB-231 (MB231) and MDA-MB-436 (MB436), *in vitro* and in *vivo*.

## Materials and Methods

### Cell culture

The human TNBC cell lines MB231 and MB436 (a gift from Columbia University, New York, USA) and their corresponding KD-DR4 and KD-DR5 cells (in which DR4 and DR5 shRNA plasmids were transfected, respectively) were maintained in RPMI-1640 media (Invitrogen, Carlsbad, USA) supplemented with 10% fetal bovine serum (FBS), 2 mM L-glutamine, 100 μg/mL streptomycin, and 100 units/mL penicillin at 37°C with 5% CO _2_ as described previously [9].

### Reagents

Primary antibodies against DR4 and β-actin were purchased from Santa Cruz Biotechnology (Dallas, USA). The other primary antibodies against caspase-8/-3, poly ADP-ribose polymerase (PARP), and DR5 were obtained from Cell Signaling Technology (Danvers, USA). HRP-conjugated secondary antibodies were provided by Proteintech (Chicago, USA).

### Screening of stably transfected cell lines with *DR4* or *DR5* knockdown

MB231 and MB436 cells were transfected with 1 μg of shRNA plasmids (DR4, DR5 or scrambled control; Santa Cruz Biotechnology) and 4 μL of transfection reagent (Santa Cruz Biotechnology) in OPTI-MEM (Gibco, Carlsbad, USA) following the instructions of the shRNA transfection protocol. The catalog number of shRNAs of DR4, DR5 and scrambled control were shown as follows: sc-35218-SH, sc-40237-SH and sc-108060. Resistance selection was carried out using puromycin antibiotic (2 μg/mL). Stably transfected cell lines containing DR4, DR5, or control shRNA plasmids were labelled KD-DR4, KD-DR5, or Ctrl, respectively.

### Cell viability assay

Cell viability was determined using the 3-(4,5-dimethylthiazol-2-yl)-5-(3-carboxymethoxyphenyl)-2-(4-sulfophenyl)-2H-tetrazolium (MTS; Promega, Madison, USA) according to the protocols provided by the manufacturer. Briefly, cells were grown in 96-well plates and incubated with varying doses of CPT (Beijing Sunbio Biotech, Beijing, China) and/or Dox (Zhejiang Hisun-Pfizer Pharmaceutical Co., Ltd., Taizhou, China) for 24 or 48 h. Optical density (OD) at 490 nm was measured using a microplate reader. The inhibition rate (%)=[1‒(average OD value of experimental group)/(average OD value of control group)]×100%.

### Drug combination studies

The 50% inhibitory concentrations (IC _50_) were calculated using nonlinear regression (log [inhibitor] vs normalized response, variable slope) using GraphPad Prism software (version 8.0.2; GraphPad, La Jolla, USA). Two different concentrations were selected for combination therapy to detect the synergistic effects. The lower limit is far below the IC _50_, and the higher limit is near the IC _50_. In a study of the antitumor effect of TRAIL combined with Dox on MCF-7 cells, the concentration ratio of TRAIL and Dox was 1:5 [10]. Considering that the antitumur effect of CPT was superior to that of TRAIL, CPT:Dox combinations were used at a fixed 1:8 ratio. MB231 and MB436 cells received various concentrations of CPT (0.004–0.063 μg/mL), Dox (0.031–0.5 μg/mL), and CPT plus Dox (at a dose ratio of 1:8) for 24 or 48 h. Based on cell viability inhibition detected by the MTS method, the combination index (CI) was calculated, and antagonism or synergism (CI>1 or CI<1) was analyzed using CalcuSyn software (Biosoft, Ferguson, USA) in drug combinations [11].

### Flow cytometry assay

After another 24 or 48 h of exposure to CPT, Dox, or CPT plus Dox, cells were harvested, centrifuged, and resuspended in binding buffer. Following the addition of phycoerythrin-conjugated Annexin V (Annexin V-PE) and 7-aminoactinomycin D (7-AAD) from BD Pharmingen Biosciences (San Diego, USA), each sample was incubated for 15 min at room temperature (RT) in the dark. Apoptosis rates were determined with a flow cytometer (FACSCanto II; BD Biosciences). The results were plotted on a graph using FlowJo software (Tree Star Inc., Ashland, USA).

### Western blot analysis

After protein concentrations were measured by BCA assay (Pierce, Rockford, USA), the whole cell lysates were subject to 12% SDS-PAGE and blotted onto polyvinylidene difluoride (PVDF) membranes (Millipore, Billerica, USA). PVDF membranes were blocked at RT for 2 h in 5% nonfat milk, probed overnight at 4°C with primary antibodies against DR4/5, caspase-8/-3, PARP, and β-actin at a 1:100–1:2000 dilution, followed by incubation with secondary antibodies (dilution 1:5000) at RT for 1 h. Finally, the protein bands were visualized using enhanced chemiluminescence solutions (Beyotime, Haimen, China).

### Xenograft model and *in vivo* therapeutic experiments

All animal studies were approved by the Institutional Animal Care and Use Committee of Bethune International Peace Hospital. Each of the 6-week-old female BALB/c nude mice was subcutaneously inoculated with 3×10 ^6^ MB231 cells in the right armpit. The tumor sizes were measured every 2–3 days. When tumor volumes reached±32 mm ^3^ (average size), as measured using the formula: tumor volume=[(length×width ^2^)/2]. Mice were randomly assigned into four groups ( *n*=5/group). The single dose and combination regimen of CPT and Dox were formulated according to the antitumor effect of CPT (15 mg/kg/d on days 1–10) combined with Dox (3 mg/kg on days 1, 5, and 9) on RPMI 8226 tumor-bearing SCID mice (data not shown). The control, CPT, Dox and CPT+Dox groups received an equal volume of normal saline (on days 1–5 and on day 9), CPT (15 mg/kg/d, on days 1–5), Dox (3 mg/kg, on days 1, 5, and 9), or CPT combined with Dox via intraperitoneal injection, respectively. Prior to study completion, nude mice remained in stable condition with no mortality observed in any group. After 21 days of treatment, all mice were sacrificed, and malignancies were resected, weighed, and embedded in paraffin for use in a terminal deoxynucleotidyl transferase (TdT)-mediated dUTP nick-end labelling (TUNEL) assay.

### TUNEL assay

According to the instructions of the colorimetric TUNEL Apoptosis Assay kit (Beyotime), apoptosis was observed in tumor tissues. Briefly, 4 μm paraffin-embedded sections were dewaxed, rehydrated with graded ethanol, and then incubated with TdT and biotin-dUTP mixtures. Afterwards, tumor tissues were counterstained with hematoxylin. Under a microscope, four random high-magnification fields were selected from each slide, and 200 cells were observed per field. The apoptosis (TUNEL-positive cells) rate was counted as follows: apoptosis rate (%)=TUNEL-positive cells/total number of cells×100%.

### Statistical analysis

All statistical analyses were performed using GraphPad Prism software (version 8.0.2). Data are presented as the mean±standard deviation (SD) of 3 independent experiments or 5 mice in each group. Data were statistically analyzed using Student’s *t* test for the comparison of two groups or one-way ANOVA followed by Tukey’s multiple comparisons test for the comparison of more than two groups. Normal distribution and homoscedasticity were checked before one-way ANOVA. *P*<0.05 was considered statistically significant.

## Results

### CPT combined with Dox synergistically inhibits the viability of MB231 and MB436 cells

**Figure 1:**
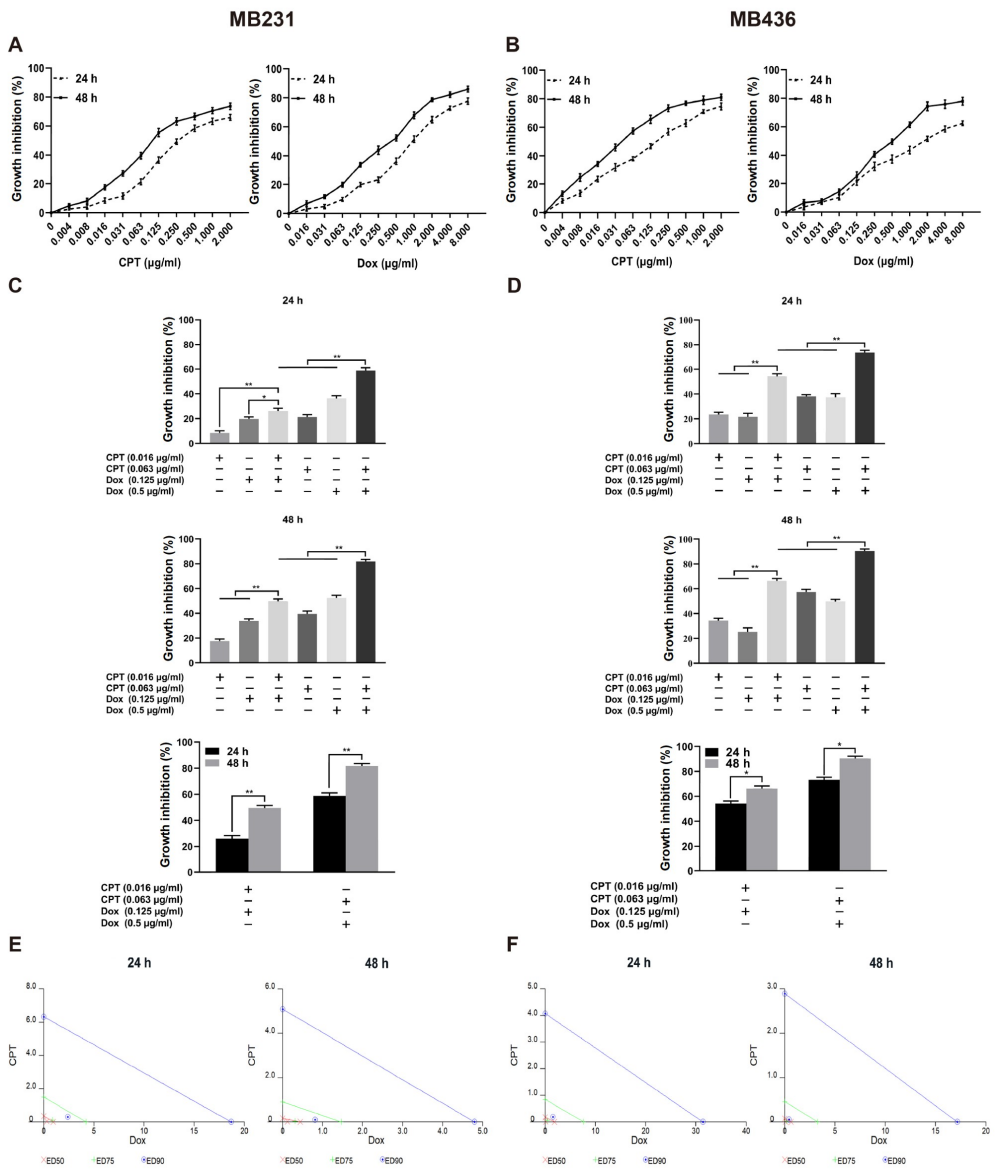
Figure 1 Effect of CPT, Dox, or a combination treatment on the viability of MDA-MB-231 (MB231) and MDA-MB-436 (MB436) cells The inhibition rates were calculated using the MTS method in MB231 (A) and MB436 (B) cells. The inhibition rates of CPT in combination with Dox in MB231 (C) and MB436 (D) cells. (E,F) The synergistic effect of CPT with Dox was analyzed using CalcuSyn software. ED50 (red line), ED75 (green line), and ED90 (blue line) represent the effective doses of drugs that inhibit cell viability at 50%, 70%, and 90%, respectively. The same color marker under the same color line indicates a synergistic effect. * P<0.05, ** P<0.01.

Both CPT and Dox decreased the viabilities of MB231 and MB436 cells in a time- and dose-dependent manner, as determined by the MTS assay ( Figure 1A,B). In MB231 cells, the IC _50_ values of CPT or Dox alone at 48 h were 0.16± 0.03 μg/mL and 0.39± 0.05 μg/mL, respectively. At 48 h, the IC _50_ values of CPT or Dox alone in MB436 cells were 0.05± 0.01 μg/mL and 0.58± 0.11 μg/mL, respectively. Further investigation indicated that in both TNBC cell lines, the combination of CPT and Dox time- and dose-dependently decreased cell viability, and the synergistic inhibitory effect of the combination treatment was significantly superior to that of the corresponding single-drug treatment ( Figure 1C–F and Tables 1, 2). These findings indicated that CPT and Dox synergistically inhibited the viability of TNBC cells.

**Table:** **Table 1** Effects of CPT and Dox combination on the growth of MB231 cells during 24 or 48 h of exposure

CPT (μg/mL)	Dox (μg/mL)	Fa		CI		Degree of synergy/antagonism
24 h	48 h		24 h	48 h		24 h	48 h
0.004	0.031	0.074	0.178		1.301	0.632		‒	**+++**
0.008	0.063	0.154	0.317		0.863	0.483		**+**	**+++**
0.016	0.125	0.287	0.516		0.594	0.348		**+++**	**+++**
0.031	0.250	0.444	0.706		0.474	0.269		**+++**	**++++**
0.063	0.500	0.613	0.835		0.376	0.224		**+++**	**++++**

CPT, circular permuted tumor-necrosis-factor-related apoptosis-inducing ligand; Dox, Doxorubicin; Fa, fraction affected by the dose (Fa represented inhibition rates of CPT combined with Dox); CI, combination index (CI>1 and CI<1 indicated antagonism and synergism, respectively). A scoring system of CI (– – – – – to + + + + + representing extremely intensive antagonism to extremely intensive synergy) refers to CalcuSyn manual (Biosoft, 2006).

**Table:** **Table 2** Combined treatment of CPT and Dox in MB436 cells during 24 or 48 h of exposure

CPT (μg/mL)	Dox (μg/mL)	Fa		CI		Degree of synergy/antagonism
24 h	48 h		24 h	48 h		24 h	48 h
0.004	0.031	0.170	0.235		0.342	0.694		**+++**	**+++**
0.008	0.063	0.357	0.414		0.174	0.366		**++++**	**+++**
0.016	0.125	0.522	0.683		0.138	0.124		**++++**	**++++**
0.031	0.250	0.644	0.834		0.139	0.065		**++++**	**+++++**
0.063	0.500	0.729	0.909		0.161	0.044		**++++**	**+++++**

CPT, circular permuted tumor-necrosis-factor-related apoptosis-inducing ligand; Dox, Doxorubicin; Fa, fraction affected by the dose (Fa represented inhibition rates of CPT combined with Dox); CI, combination index (CI>1 and CI<1 indicated antagonism and synergism, respectively). A scoring system of CI (– – – – – to + + + + + representing extremely intensive antagonism to extremely intensive synergy) refers to CalcuSyn manual (Biosoft, 2006).

### CPT combined with Dox induces synergistic apoptosis in TNBC cells

**Figure 2:**
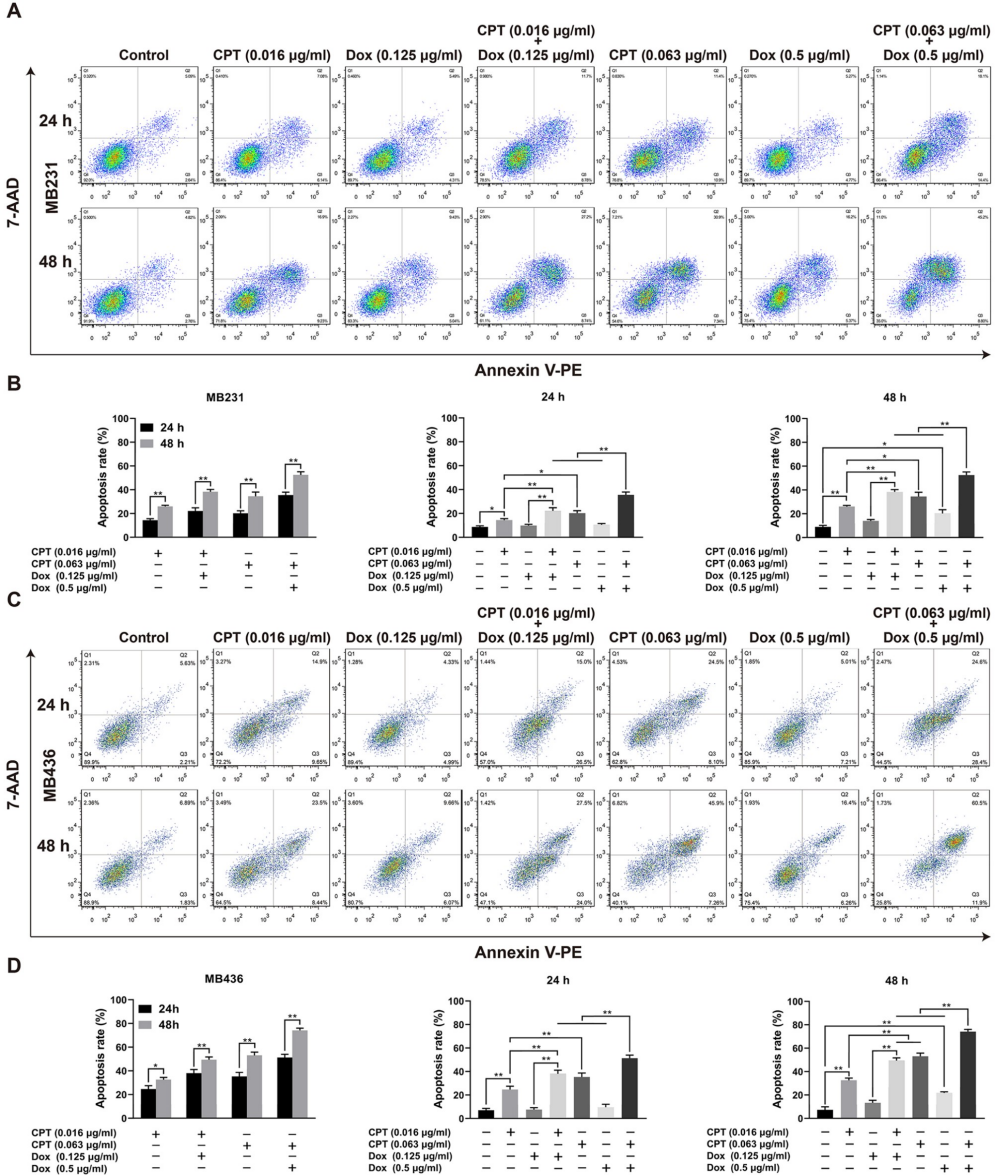
Figure 2 Effects of CPT and Dox alone or in combination on the apoptosis of MDA-MB-231 (MB231) and MDA-MB-436 (MB436) cells MB231 (A,B) and MB436 (C,D) cells were labelled with Annexin V-PE/7-AAD, and their apoptosis was detected by flow cytometry. * P<0.05, ** P<0.01.

After exposure to CPT for 24 or 48 h in MB231 ( Figure 2A,B) and MB436 ( Figure 2C,D) cells, more cells underwent either early (Annexin V-PE ^+^/7-AAD ^−^) or late (Annexin V-PE ^+^/7-AAD ^+^) apoptosis in a time- and dose-dependent manner. Additionally, apoptosis was increased only after 48 h of Dox (0.5 μg/mL) treatment at the different time points and doses tested. More importantly, CPT plus Dox triggered more apoptosis than monotherapy in a time- and dose-dependent manner in MB231 and MB436 cells, suggesting that the CPT and Dox combination triggered synergistic apoptosis in TNBC cells.

### Activation of caspases-8/-3 and proteolytic cleavage of PARP in TNBC cells is enhanced by treatment with CPT plus Dox

**Figure 3:**
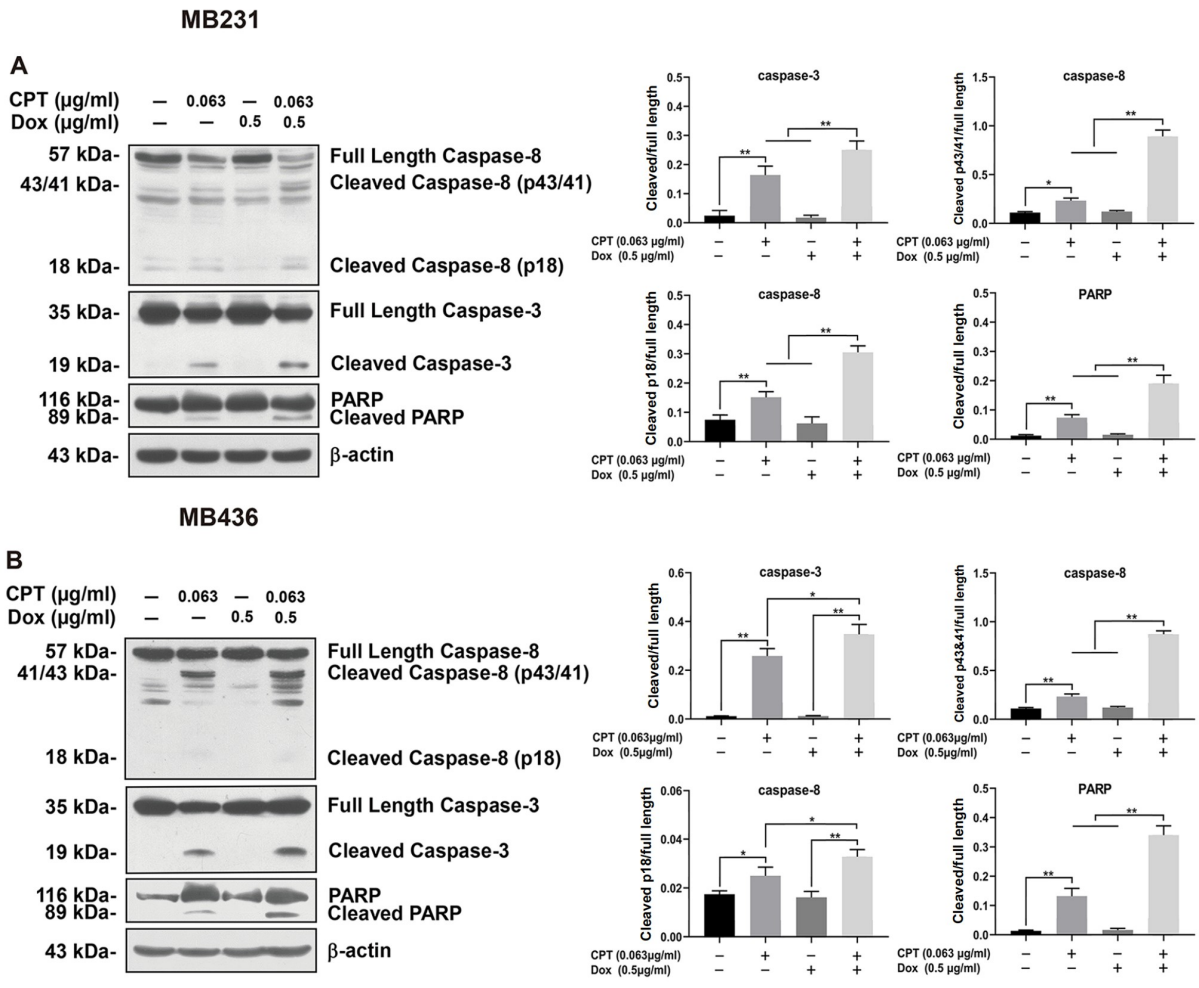
Figure 3 Effect of CPT, Dox or CPT plus Dox on the protein levels of cleaved caspases-8/-3 and PARP in MDA-MB-231 (MB231) and MDA-MB-436 (MB436) cells The two cell lines were incubated with or without CPT, Dox or their combination for 24 h. The expressions of caspases-8/-3, PARP, and their cleaved counterparts were detected by western blot analysis in MB231 (A) and MB436 (B) cells. β-Actin served as a loading control. * P<0.05, ** P<0.01.

The levels of cleaved caspase-8/-3 and PARP were considerably elevated in both TNBC cells treated with CPT alone and in combination with Dox for 24 h compared to the control. Cleaved caspases-8/-3 and PARP levels were markedly higher with CPT plus Dox than with either single agent. Dox monotherapy did not enhance caspases-8/-3 or PARP cleavage over the control ( Figure 3A,B). These results showed that CPT plus Dox induced caspase-dependent apoptosis in TNBC cells.

### CPT triggers apoptosis through DR4 and DR5 simultaneously

**Figure 4:**
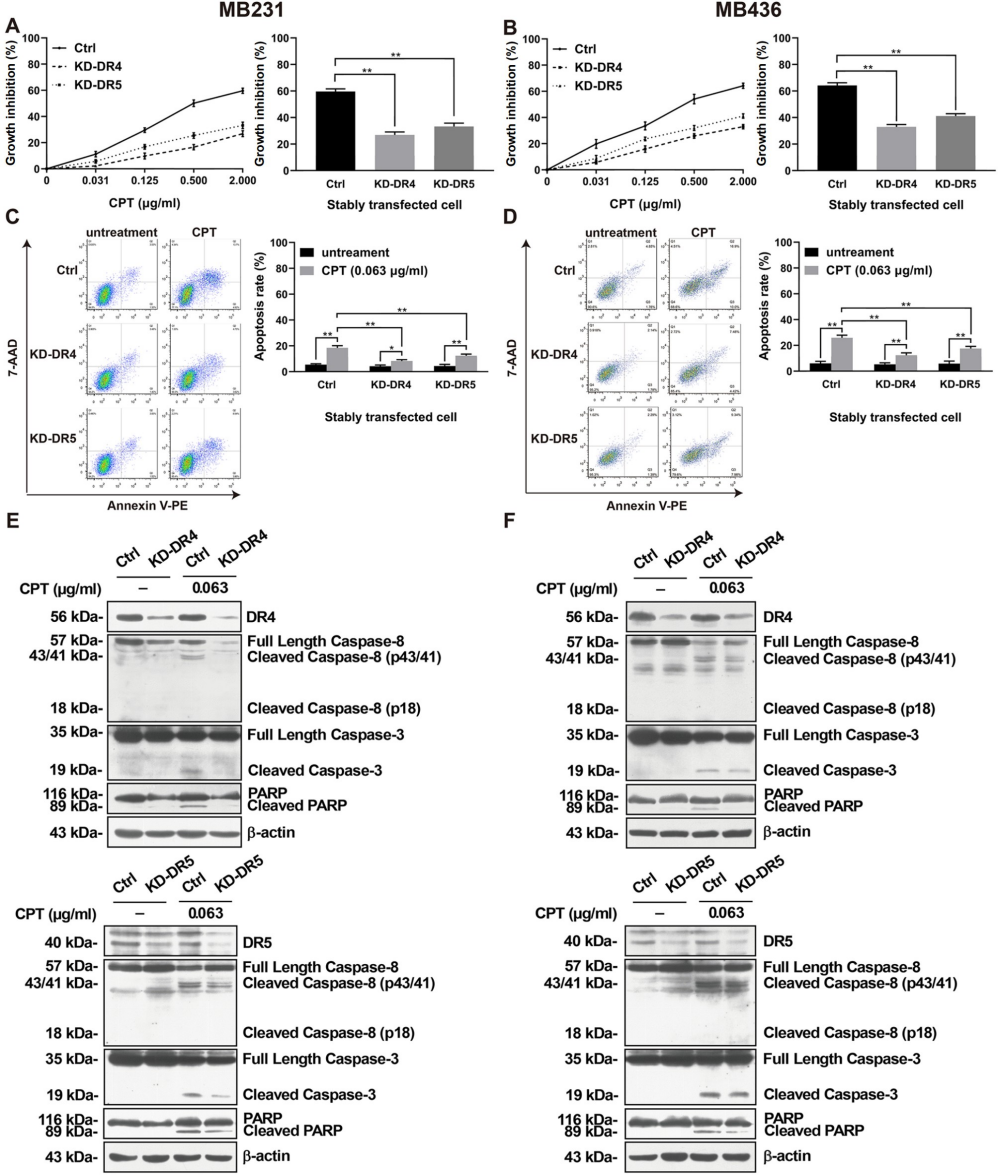
Figure 4 Effect of knockdown of *DR4* or *DR5* on CPT-induced growth inhibition, apoptosis, and apoptosis-related proteins in MDA-MB-231 (MB231) and MDA-MB-436 (MB436) cells Ctrl and KD-DR4/5 cells were treated for 24 h with or without CPT. (A,B) The cell growth inhibition rate was detected by the MTS method. (C,D) The percentage of apoptosis was detected by flow cytometry. (E,F) The expressions of caspases-8/-3, PARP, and their cleaved counterparts were detected by western blot analysis. β-Actin served as a loading control. * P<0.05, ** P<0.01.

Knockdown (KD) of either *DR4* or *DR5* reduced CPT toxicity across a wide concentration range ( Figure 4A,B). Compared with Ctrl cells, the inhibition rate caused by 2 μg/mL CPT for 24 h was distinctly reduced in KD-DR4 and KD-DR5 MB231 and MB436 cells ( Figure 4A,B). After treatment with CPT (0.063 μg/mL) for 24 h, the apoptosis rates ( Figure 4C,D) and levels of cleaved caspases-8/-3 and PARP ( Figure 4E,F) were lower in KD-DR4 and KD-DR5 cells than in Ctrl cells. These results suggested that both DR4 and DR5 played proapoptotic roles in CPT-induced apoptosis of TNBC cells.

### Dox upregulates DR4 and DR5 expressions

**Figure 5:**
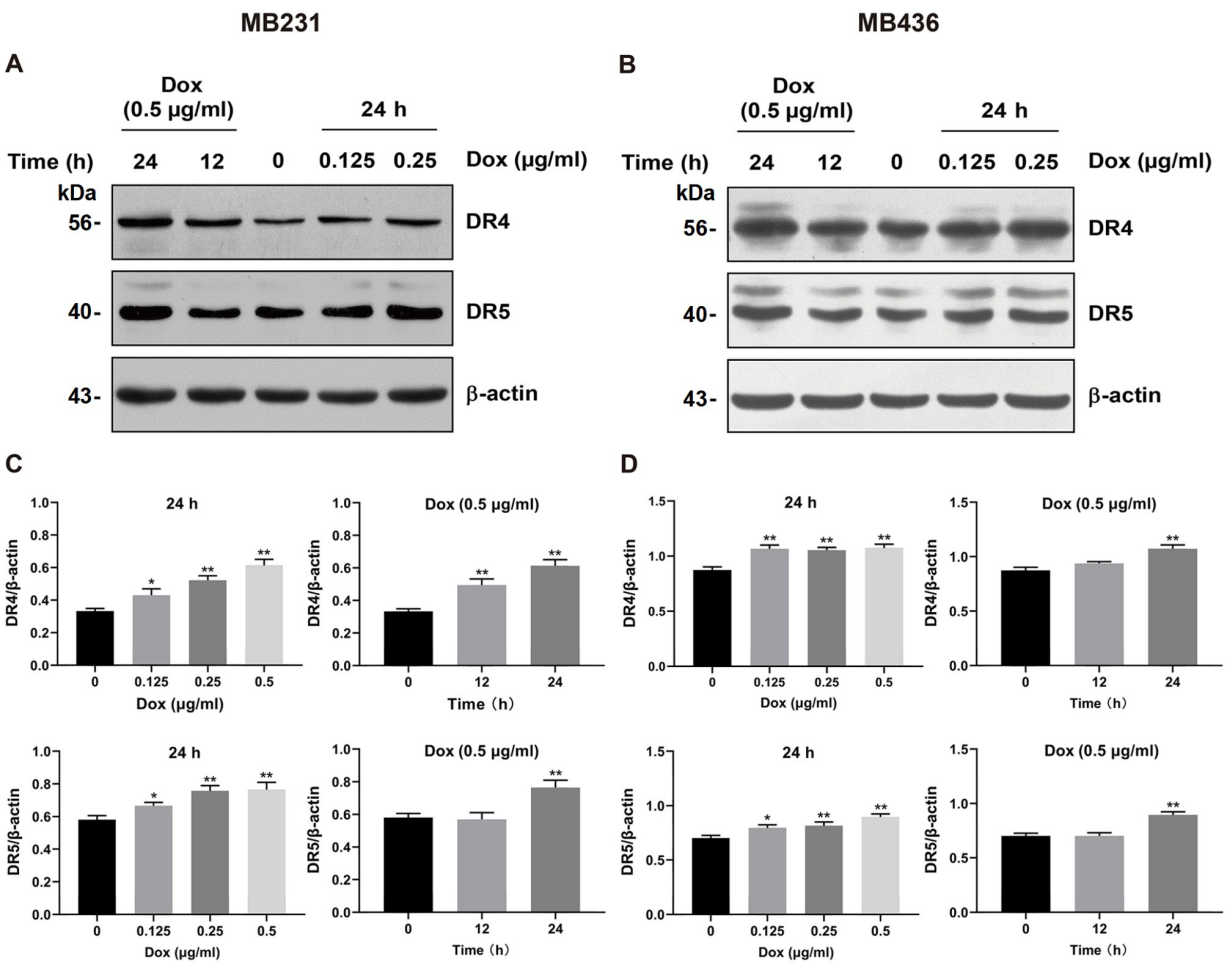
Figure 5 Dox upregulates DR4 and DR5 expressions in MDA-MB-231 (MB231) and MDA-MB-436 (MB436) cells (A,B) DR4 and DR5 expressions were measured by western blot analysis, where β-actin was used as a loading control. (C,D) The relative expression intensity of DR4/5 was quantified using ImageJ software in MB231 (C) and MB436 (D) cells. * P<0.05, ** P<0.01 versus untreated cells.

The expressions of DR4 and DR5 were measured by western blot analysis. DR4 and DR5 were both notably augmented in MB231 and MB436 cells after receiving Dox at different concentrations and exposure time points ( Figure 5A,B). Each treatment group was compared with the untreated group. Different doses of Dox (0.125, 0.25, and 0.5 μg/mL) increased DR4 and DR5 expressions, and the most obvious effect was observed at 0.5 μg/mL Dox in both TNBC cell lines. Moreover, 0.5 μg/mL not only increased DR4 level in MB231 cells after 12 and 24 h but also dramatically boosted DR5 expression in MB231 cells and MB436 cells at 24 h ( Figure 5C,D). These results implied that Dox upregulated the expressions of both DRs in a dose-dependent manner.

### CPT plus Dox inhibits growth and enhances apoptosis in MB231 xenografts

**Figure 6:**
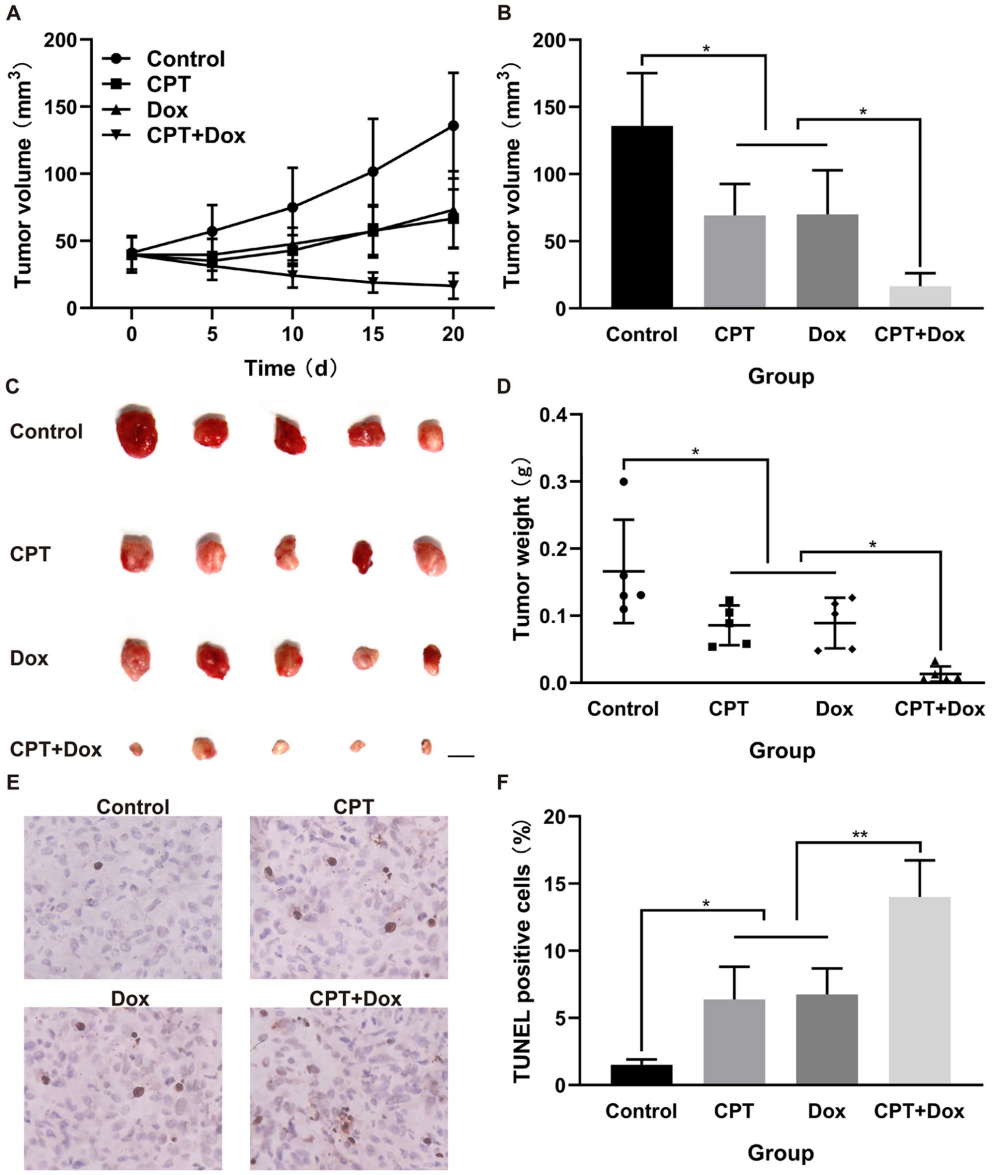
Figure 6 CPT combined with Dox reduces the growth of MDA-MB-231 (MB231) xenografts in nude mice (A,B) Tumor volumes were measured, and tumor growth curves were plotted for the four groups ( n=5/group). (C,D) Tumor tissues were resected and weighed in each group. Scale bar: 0.5 cm. (E,F) The percentage of apoptotic cells was measured by the TUNEL assay. * P<0.05, ** P<0.01.

As shown in Figure 6A, CPT and Dox alone or in combination suppressed tumor growth in nude mice, which was consistent with the findings *in vitro*. Tumor volumes and weights were markedly lower with combination treatment than with either single agent ( Figure 6B–D). The TUNEL assay showed that CPT combined with Dox induced a significant increase in apoptosis in MB231 cell xenografts ( Figure 6E,F). These results revealed that CPT and Dox inhibited the growth and enhanced the apoptosis of MB231 cells *in vivo*.

## Discussion

Owing to the lack of available receptor targets, TNBC patients continue to be primarily treated with chemotherapy. However, the complete pathological response rate of standard anthracycline multidrug combination therapy is only 20–40% [12]. In the current study, we tried a new therapeutic strategy using CPT combined with low-dose Dox to treat TNBC cells and observed their efficacy *in vitro* and *in vivo*. CPT can inhibit the viability of MB231 and MB436 cells. CPT further shows a synergistic inhibitory effect in combination with low-dose Dox, which is mainly achieved by promoting apoptosis. Subsequently, *in vivo* experiments of tumor-bearing nude mice also demonstrated that CPT and Dox alone or in combination inhibited tumor growth, with the smallest and lightest tumor volume and tumor weight in the combined treatment group, indicating that CPT inhibits the growth of MB231 cells in nude mice and has stronger antitumor activity in combination with low-dose Dox. The results of the TUNEL assay on tumor tissues revealed that the antitumor effects of CPT and its combination with Dox were related to apoptosis induction in tumor cells. These findings implied that the combination of CPT with low-dose Dox can achieve significant synergistic antitumor activity while decreasing the risk of dose-dependent toxicities associated with Dox.

In addition to the side effects of Dox, there are resistance problems that limit Dox treatment for TNBC. Resistance can occur either during treatment or naturally unresponsive to chemotherapy. Clinically, TNBC patients have a poor prognosis owing to a more aggressive phenotype, chemotherapy resistance and lack of targeted gene therapy [13]. There is growing evidence that TRAIL-induced apoptosis may have therapeutic potential in TNBC [14]. TRAIL can induce apoptosis via an external pathway in which death ligands bind to DRs, recruit and activate caspase-8, a caspase that initiates apoptosis, and then activate the apoptosis executor caspase-3 to induce apoptosis [4]. In addition, conventional chemotherapeutic agents have been observed to enhance TRAIL-triggered apoptosis by enhancing the caspase cascade [15]. Previous studies have shown that CPT/5-fluorouracil combination therapy activates caspases-8/-3 and promotes apoptosis in SW480 and HCT116 cells [16]. To investigate whether the caspase cascade pathway is critical for apoptosis mediated by CPT plus Dox, we analyzed caspase expression by western blot analysis. The results showed that cleaved caspases-8/-3 levels were elevated in both MB231 and MB436 cells receiving CPT or CPT plus Dox, but this elevation was more pronounced after combined treatment. PARP, a substrate for DNA repair enzymes and caspase-3, is associated with apoptosis. PARP cleavage was increased in the CPT and CPT plus Dox groups, but especially in the cotreatment group. Our data suggested that the enhanced antitumor mechanism of CPT plus Dox in TNBC is associated with the exogenous apoptotic pathway.

Binding of CPT to DRs is a key step in cell apoptosis. MB231 and MB436 cells express two DRs that promote cell apoptosis. Therefore, it is necessary to investigate whether CPT binds to DR4 or DR5 to induce cell apoptosis. Our data showed that when *DR4* or *DR5* was knocked down, CPT’s capacity to reduce cell viability and promote apoptosis was diminished, and caspases-8/-3 and PARP fragments were significantly reduced, indicating that CPT acts by binding to DR4 and DR5 in MB231 and MB436 cells. The findings are not entirely consistent with those reported by Rahman *et al*. [17]. In their investigation, *DR4* knockdown had only a slight impact on cytotoxicity [17]. These data implied that different TRAILs have different affinities for DR4 and DR5 because of their unique protein structures. Not all TNBC cells express DR4 and DR5, and some TNBC cells express only one of them [17]. CPT has the ability to bind to both DR4 and DR5; thus, it has broad potential applications. DRs play key roles in CPT-induced cell apoptosis. The effect of Dox on DR4 and DR5 expressions was also the focus of our work. Two previous studies on the influence of Dox on DR expression in TNBC cells have yielded inconsistent findings. Keane *et al*. [18] demonstrated that Dox could not increase DR4 and DR5 expressions in MB231 cells. Another study using immunofluorescence staining found that Dox enhanced DR4 and DR5 expressions on the membrane surface of MB231 cells [19]. The conclusion of the latter study was consistent with our results. The discrepancy between the two previous studies may be due to differences in experimental methods. Additionally, previous studies reported that MB231 and MB436 cells with high DR5 expression exhibited enhanced sensitivity to TRAIL, while the TNBC cell lines BT20 and HCC1937 with low DR4 and DR5 expressions were resistant to TRAIL but sensitive to TRAIL when DR4 and DR5 expression levels were increased [14]. We also obtained similar results, where elevated levels of DR4 and DR5 expressions led to an increase in the sensitivity of MB-231 and MB-436 cells to CPT. These results suggested that upregulation of DR expression by Dox is one of the molecular mechanisms by which CPT and Dox exert synergistic antitumor effects. Given that DR4 and DR5 are expressed in the majority of breast malignancies and sometimes even overexpressed [20], the combination of CPT and Dox may have promising applications in breast cancer treatment. Since the TNBC cell lines used here exhibited a mesenchymal phenotype, it will be important to determine if similar responses occur in cell lines with an epithelial phenotype. Additionally, future studies should examine whether CPT-induced apoptosis involves Bcl-2 family members. In subsequent studies, more detail should be done to address these concerns.

In summary, our findings demonstrated that CPT can induce apoptosis in TNBC cells (MB231 and MB436) by interacting with both DR4 and DR5. Furthermore, CPT combined with Dox has a synergistic antitumor effect, the mechanism of which is partly dependent on the Dox-induced upregulation of DR4 and DR5 expressions. This study suggested that CPT combined with low-dose Dox may be a feasible therapeutic strategy to synergistically kill tumor cells, as well as to prevent the possible development of cardiotoxicity associated with the cumulative dose of Dox. These findings provide a basis for clinical trials of the cotreatment of CPT and Dox for patients with TNBC.
